# Crossing fibers may underlie the dynamic pulling forces of muscles that attach to cartilage at the tip of the nose

**DOI:** 10.1038/s41598-023-45781-1

**Published:** 2023-11-02

**Authors:** Mi-Sun Hur, Seunggyu Lee, Han-Sung Jung, Richard A. Schneider

**Affiliations:** 1 Department of Anatomy, Daegu Catholic University School of Medicine, Daegu, Korea; 2 Division of Applied Mathematical Sciences, Korea University, Sejong, Korea; 3 Biomedical Mathematics Group, Institute for Basic Science, Daejeon, Korea; 4 Division in Anatomy and Developmental Biology, Department of Oral Biology, Taste Research Center, BK21 FOUR Project, Oral Science Research Center, Yonsei University College of Dentistry, Seoul, Korea; 5 Department of Orthopaedic Surgery, University of California at San Francisco, 513 Parnassus Avenue, S-1161, San Francisco, CA 94143-0514 USA

**Keywords:** Musculoskeletal system, Medical imaging, Anatomy

## Abstract

The present study used microdissection, histology, and microcomputed tomography (micro-CT) with the aims of determining the prevalence and patterns of the depressor septi nasi (DSN) and orbicularis oris (OOr) muscles attached to the footplate of the medial crus (fMC) of the major alar cartilage, focusing on their crossing fibers. The DSN and OOr attached to the fMC of the major alar cartilage were investigated in 76 samples from 38 embalmed Korean adult cadavers (20 males, 18 females; mean age 70 years). The DSN, OOr, or both were attached to the fMC. When the DSN ran unilaterally or was absent, some OOr fibers ascended to attach to the fMC instead of the DSN in 20.6% of the samples. Crossing fibers of the DSN or OOr attached to the fMC were found in 82.4% of the samples. Bilateral and unilateral crossing fibers were found in 32.4% and 50.0%, respectively, and no crossing fibers were found in 17.6%. The DSN and OOr that attached to the fMC could be categorized into six types according to presence of the DSN and the crossing patterns of the DSN and OOr. Anatomical findings of the DSN and OOr that attached to the fMC were confirmed in histology and micro-CT images. These findings offer insights on anatomical mechanisms that may underlie the dynamic pulling forces generated by muscles that attach to the fMCs and on evolutionary variation observed in human facial expressions. They can also provide useful information for guiding rhinoplasty of the nasal tip.

## Introduction

The depressor septi nasi (DSN) plays important roles in the dynamics and rhinoplasty of the nasal tip^1–4^. The DSN is described in the literature as a nasal depressor muscle or digastricus nasi-septi labialis muscle because of its dynamics in facial expressions, especially when smiling^5^. DSN overactivity induces a smiling deformity characterized by a descending nasal tip, shortened upper lip, and transverse crease in the mid-philtral area^6^. Ohtsuka^7^ also reported a case of DSN hypertrophy that caused columella deformity.

Several clinical studies have emphasized the importance of DSN resection during rhinoplasty in patients with DSN hyperactivity using various surgical techniques^3,6^. Rowe-Jones and van Wyk^8^ found that a strong DSN influences the posterior septal angle and that dividing the DSN reduces this angle. Rohrich et al.^6^ found that dissection and transposition (not resection) of both paired DSNs during rhinoplasty improved or corrected nasal tip–upper lip imbalance and increased the fullness of the central upper lip.

Releasing pulling forces of the muscles that affect the nasal tip during rhinoplasty requires an understanding of the detailed anatomy of attachments and arrangements of the muscles. The DSN also plays an important role in the external nasal valve, nasal respiration, and movement mimicking^4^. The DSN therefore requires a careful surgical approach to improve its aesthetic and functional outcomes.

Orbicularis oris (OOr) and DSN muscles influence smiling deformities. Benlier et al.^9^ found that DSN repositioning improved mild cases of smiling deformities, but also modifying the OOr improved moderate and severe cases. These authors also found that the OOr was responsible for lip shortening and the transverse crease in the mid-philtral area. Benlier et al.^10^ proposed that modifying both the DSN and OOr should be considered as a part of aesthetic rhinoplasty.

Several studies have found that the DSN is attached to or interspersed with the OOr^4–6,11^. However, details on the crossing fibers of the DSN or OOr that attach to the footplate of the medial crus (fMC) have not been reported in the literature. Furthermore, in cases where the DSN is absent or rudimentary, there are no descriptions as to whether the OOr fibers attach to the fMCs instead of the DSN.

When the crossing fibers of the DSN or OOr are attached to the fMC, their length and direction can differ from those of the DSN fibers. If the crossing fibers are unilaterally dominant, asymmetric movements can occur in the nasal tip, further producing imbalance between the nose and upper lip. Dynamic forces from contracting muscles that attach to the fMC should therefore be considered using vectors of the crossing fibers.

Various authors have defined the DSN differently. The myrtiformis was originally divided into inner (medial) fibers, which ran to the mobile septum as the DSN, and outer (lateral) fibers, which surrounded the nostril base as the depressor alae nasi (DAN) muscle^12^. However, some authors have reported that the DSN included the DAN^1,4,13^. In the present study, the DSN was defined as the muscle attached to the fMC or nasal septum and was different from the DAN.

In the present study, we used microdissection, histology, microcomputed tomography (micro-CT), and numerical simulations to determine the prevalence and patterns of the DSN and OOr attached to the fMC of the major alar cartilage. We focused especially on their crossing fibers. Our results offer insights on anatomical mechanisms that may underlie the dynamic pulling forces generated by muscles that attach to the fMCs, and our findings provide useful information for rhinoplasty involving the modification or repositioning of muscle fibers that attach to the fMC. An understanding of the mechanism of dynamic pulling forces of the muscles that are attached to the fMC based on numerical simulations can also help evaluate preoperative muscle contractions and prevent postoperative deformities caused by muscle hyperactivity.

## Results

### Macroscopic anatomy of the muscle fibers attached to the fMCs: DSN and OOr

The DSN, OOr, or both were attached to the fMC. The DSN was present bilaterally in 24 of 34 cadavers (70.6%) and unilaterally in 6 (17.6%). The DSN was bilaterally absent in four cadavers (11.8%). When the DSN was present unilaterally or was absent, some OOr fibers ascended to attach to the fMCs instead of the DSN in 14 of 68 samples (20.6%).

When present, the DSN originated from the maxilla periosteum above the central and lateral incisors with the DAN. The DSN ascended curving superomedially and anteriorly, while the DAN ascended superiorly to attach to the vestibular skin just above the nostril sill. The DSN or some OOr fibers were attached to the base and posterior surface of the fMC, but sometimes also to its adjacent vestibular skin or membranous septum. The DSN was usually attached to the lateral base of the corresponding fMC, and some DSN or OOr fibers and their crossing fibers were attached to the medial base or posterior surface of the fMC. When the angle between fMCs was narrower, the DSN or some OOr fibers ascended more anteriorly to attach to the fMCs.

**Figure 1:**
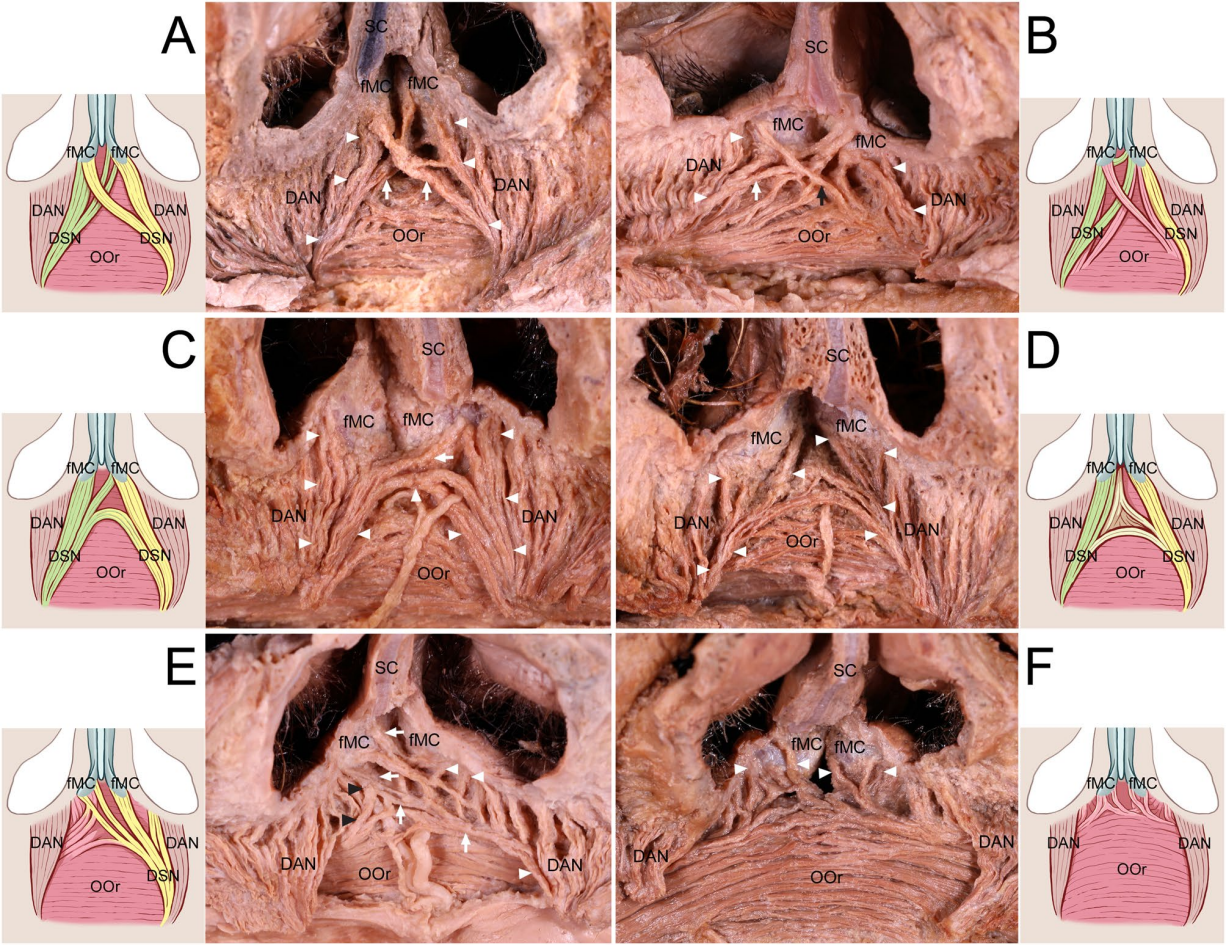
Crossing patterns of the depressor septi nasi (DSN) and orbicularis oris (OOr) muscles attached to the footplates of the medial crura (fMCs). (**A**) Type I: Both DSNs (arrowheads) were predominantly attached to the corresponding fMCs, and some of their fibers (arrows) crossed to attach to the fMCs on the contralateral sides as bilateral crossing fibers. (**B**) Type II: Both DSNs (arrowheads) were predominantly attached to the corresponding fMCs. Some DSN fibers (white arrow) on one side and some OOr fibers (black arrow) on the contralateral side crossed each other to attach to the fMCs as bilateral crossing fibers. (**C**) Type III: Both DSNs (arrowheads) were predominantly attached to the corresponding fMCs, and some DSN fibers (arrow) on one side crossed to attach to the fMC on the contralateral side as unilateral crossing fibers. Connecting fibers between both DSNs beneath the fMCs were often found in an arched shape in this type. (**D**) Type IV: The DSNs (arrowheads) were present bilaterally and there were no crossing fibers of the DSN or OOr attached to the fMCs. (**E**) Type V: The DSN (white arrowheads) on one side and some OOr fibers (black arrowheads) on the contralateral side were predominantly attached to the corresponding fMCs. Some of the DSN (arrows) on one side crossed to attach to the fMC on the contralateral side as unilateral crossing fibers. (**F**) Type VI: When the DSN was not present, some OOr fibers (arrowheads) ascended to attach to the fMCs instead of the DSNs. *DAN* depressor alae nasi, *SC* septal nasal cartilage.

In 28 of 34 cadavers (82.4%), we observed crossing fibers of the DSN or OOr attached to the fMCs, which were bilateral in 11 cadavers (32.4%) and unilateral in 17 (50.0%). The DSN and OOr attached to the fMCs could be categorized into the following six patterns according to presence of the DSN and crossing patterns of the DSN and OOr (Fig. 1):

1. Type I (*n* = 6 of 34 cadavers, 17.6%, Fig. 1A), in which the DSN was present bilaterally and both DSNs were predominantly attached to their corresponding fMCs. Some fibers of both DSNs crossed each other to attach contralaterally to the fMCs as bilateral crossing fibers. In two such cadavers, some OOr fibers on one side also crossed the midline to attach to the fMC on the contralateral side with the crossing fibers of the DSN.
2. Type II (*n* = 5, 14.7%, Fig. 1B), in which the DSN was present bilaterally and both DSNs were predominantly attached to their corresponding fMCs. Some DSN fibers on one side and some OOr fibers on the contralateral side crossed each other to attach to the fMCs as bilateral crossing fibers.
3. Type III (*n* = 10, 29.4%, Fig. 1C), in which the DSN was present bilaterally and both DSNs predominantly attached to their corresponding fMCs. Some DSN fibers on one side crossed to attach to the fMC on the contralateral side as unilateral crossing fibers. In two such cadavers, some OOr fibers crossed together along with the crossing fibers of the DSN or were attached to the corresponding fMC.
4. Type IV (*n* = 3, 8.8%, Fig. 1D), in which the DSN was present bilaterally and the DSNs and OOrs had no crossing fibers that attached to the fMCs.
5. Type V (*n* = 6, 17.6%, Fig. 1E), in which the DSN was present unilaterally. The DSN on one side and some OOr fibers on the contralateral side were predominantly attached to their corresponding fMCs. Some DSN or OOr fibers on one side crossed to attach to the fMC on the contralateral side as unilateral crossing fibers.
6. Type VI (*n* = 4, 11.8%, Fig. 1F), in which the DSN was absent bilaterally. Instead, some OOr fibers beneath the fMCs ascended to attach to the fMCs. In one cadaver, some OOr fibers crossed the midline and were attached to the fMC on the contralateral side.

Fibers connecting between both DSNs or between the DSN and OOr that attached to the fMCs were found in an arched shape in 11 cadavers (32.4%) and 1 cadaver (2.9%), respectively. The connecting fibers were observed more in Type III with unilateral crossing fibers attached to the fMC than in the other types. The OOr fibers were attached to the fMCs instead of the DSN in Type VI. The number of connecting fibers was usually approximately one-third of that in the DSN. A few DSN, OOr, or connecting fibers between the DSNs coursed anteriorly toward the nasal tip in three samples. Some medial fibers of the DSN blended with the deep fibers of the upper OOr in 22 cadavers (64.7%).

### Arrangements of the DSN and OOr adjacent to the nasal base in micro-CT images with the corresponding dissected specimen

**Figure 2:**
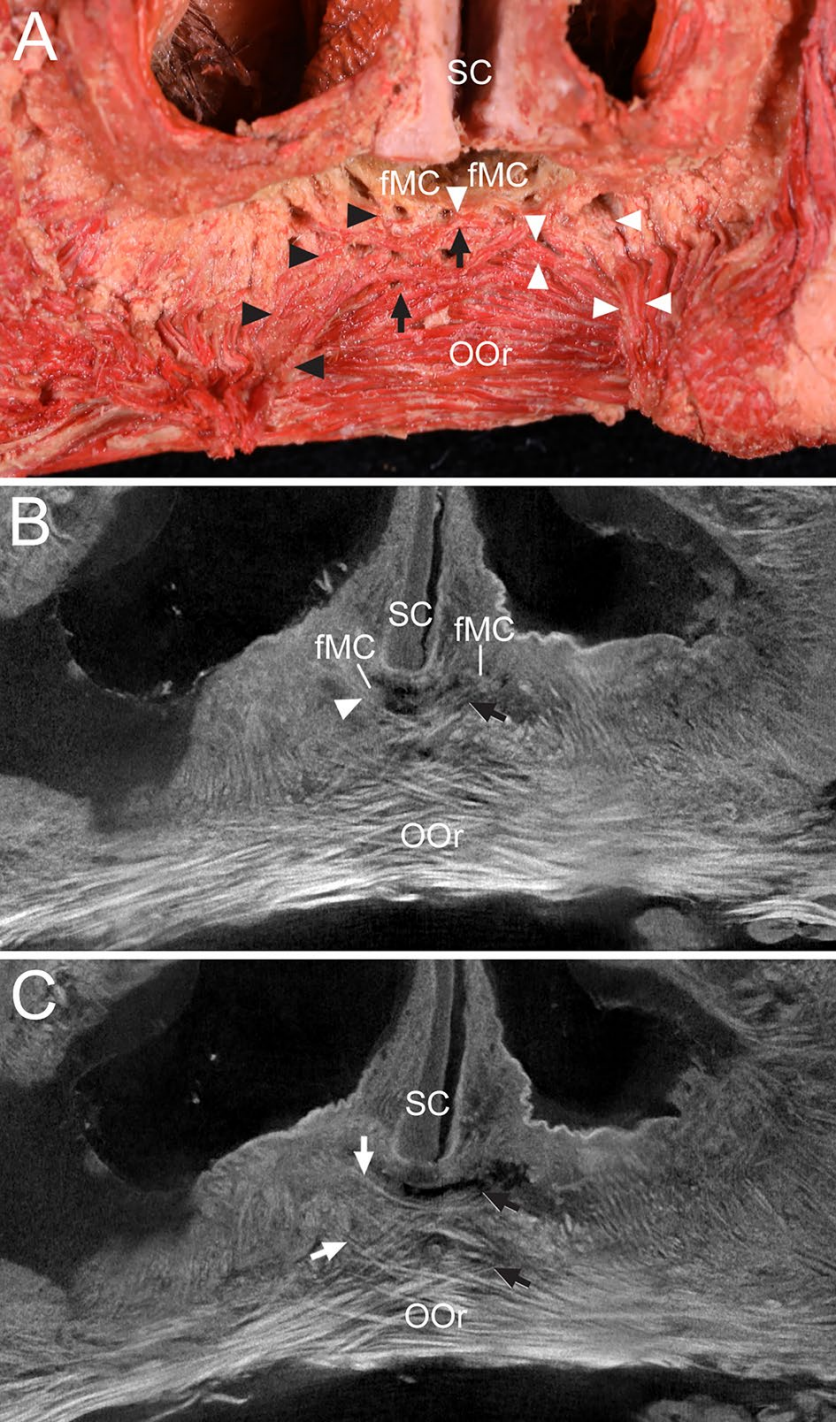
Crossing fibers of the DSN and OOr attached to the fMCs and their adjacent vestibular skin in the medial upper lip on dissection and microcomputed tomography (micro-CT) images with corresponding dissected specimen in the coronal plane. (**A**) In the dissected specimen at the posterior aspect, the left DSN (black arrowheads) was attached to the left fMC and the right DSN (white arrowheads) was attached to the right and left fMCs. Some OOr fibers (arrows) on the left side crossed to attach to the vestibular skin adjacent to the right fMC. (**B**) Micro-CT image at the anterior aspect showing that some right DSN fibers (arrowhead) crossed to attach to the left fMC, and some OOr fibers (arrow) on the left side crossed the midline to attach to the right fMC. (**C**) Micro-CT image showing that some OOr fibers (white arrows) on the right side and some OOr fibers (black arrows) on the left side crossed the midline at a more anterior site to attach to the vestibular skin adjacent to the fMC on their contralateral sides. Serial images of the crossing fibers of the DSN are presented in a Supplementary Video S1.

Serial micro-CT scans revealed the arrangements and courses of the DSN and OOr adjacent to the fMCs, and they were identified using the anatomical observation of the same specimen (Fig. 2 and Supplementary Video S1). In the corresponding dissected specimen, the arrangements and courses of the DSN and OOr fibers were confirmed on serial micro-CT images. The muscle fibers adjacent to the fMCs and the fibers crossing toward the fMCs were observed in the coronal plane. In the medial upper lip adjacent to the fMCs, several crossing fibers of the DSN and OOr were attached to the fMCs and their adjacent vestibular skin on the contralateral sides.

### Crossing fibers of the DSN in micro-CT images before histological sectioning

**Figure 3:**
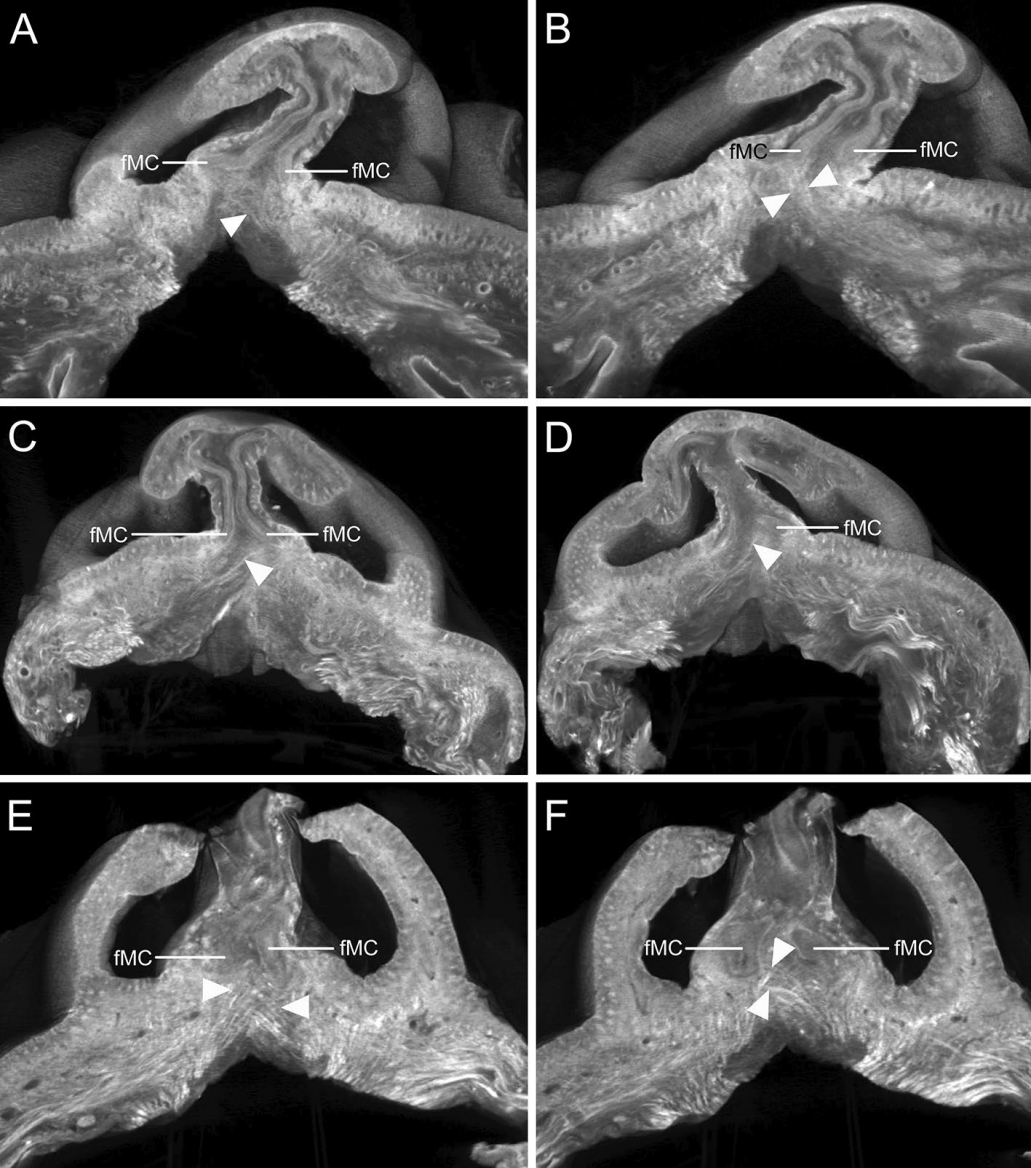
Micro-CT images of the crossing fibers of the DSN (arrowheads) in the plane parallel to the nostrils. (**A** and **B**) Some crossing fibers of the left DSN appeared to attach to the fMC on the right side. (**C** and **D**) Some crossing fibers of the right DSN appeared to attach to the fMC on the left side. Serial images of the crossing fibers of the DSN in the plane parallel to the nostrils are presented in the supplementary videos. (**E**) Crossing fibers of both the DSNs appeared to attach to the fMCs on the contralateral sides. (**F**) At deeper sites, some crossing fibers of the left DSN appeared to attach to the fMC on the right side. Serial images of the crossing fibers of the DSN are presented in Supplementary Videos S2–S4.

The muscle fibers adjacent to the fMCs were traced on the planes parallel to the nostrils using serial micro-CT images (Fig. 3 and Supplementary Videos S2–S4). Since the DSN ascended superomedially and anteriorly, its crossing fibers were clearly observed in the anterior oblique plane. Some crossing fibers of the DSN appeared to attach to the fMC on the contralateral side. The number of crossing fibers varied among the samples.

### Microscopic anatomy of the muscle fibers attached to the fMCs

**Figure 4:**
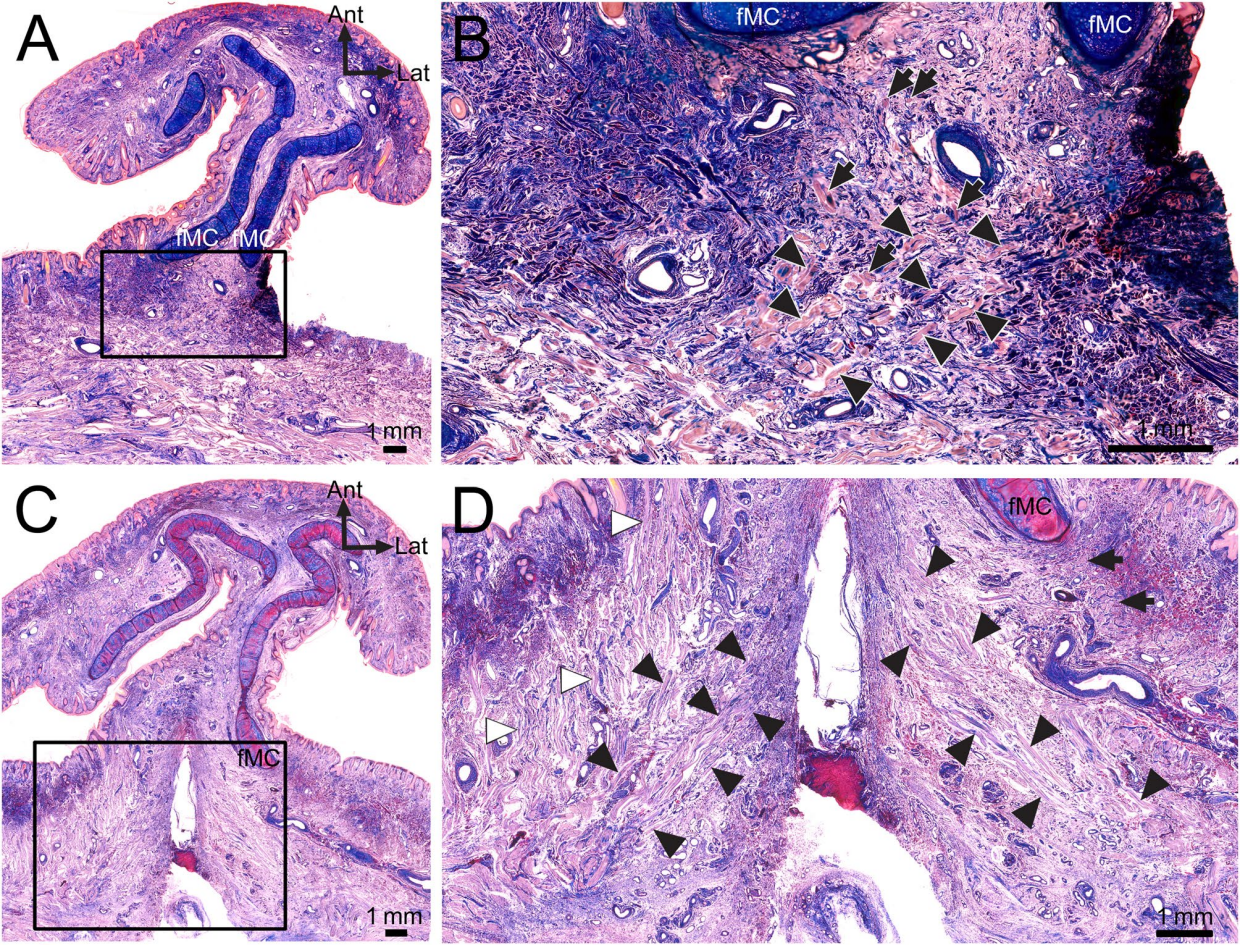
Transverse sections of histological examinations of the muscle fibers attached to the fMCs. (**A**) In a transverse section at the distal aspect of the fMCs, some muscle fibers were found just posterior to the fMCs. (**B**) Enlarged view of the rectangular area in panel A. Some muscle fibers crossing each other were found at the posterior site between the fMCs. Since the crossing fibers ascended obliquely and superomedially toward the fMC on the contralateral side, the crossing fibers just posterior to fMCs appeared as cut fibers. Arrowheads indicate the crossing fibers of the left DSN toward the right fMC. Arrows indicate the crossing fibers of the right DSN toward the left fMC. (**C**) In a superior transverse section of the fMCs of the nose, some muscle fibers of the left and right DSNs coursed obliquely and medially. (**D**) Enlarged view of the rectangular area in panel C. Some lateral muscle fibers (arrows) of the right DSN were located adjacent to the posterolateral aspect of the right fMC. Some medial muscle fibers of the left and right DSNs (black arrowheads) coursed obliquely and medially. The DAN (white arrowheads) was located just lateral to the DSN, attached to the vestibular skin. *Ant* anterior, *Lat* lateral.

In transverse sections of the nose obtained at several levels of the fMCs, muscle fibers attached to the fMCs and their adjacent muscle fibers were usually observed at the posterior and posterolateral aspects of both fMCs (Fig. 4). At the site posterior to both fMCs, some crossing fibers coursed toward the fMC on the contralateral side. These crossing fibers of the DSN were also observed in the transverse histological sections after performing micro-CT of the same samples.

**Figure 5:**
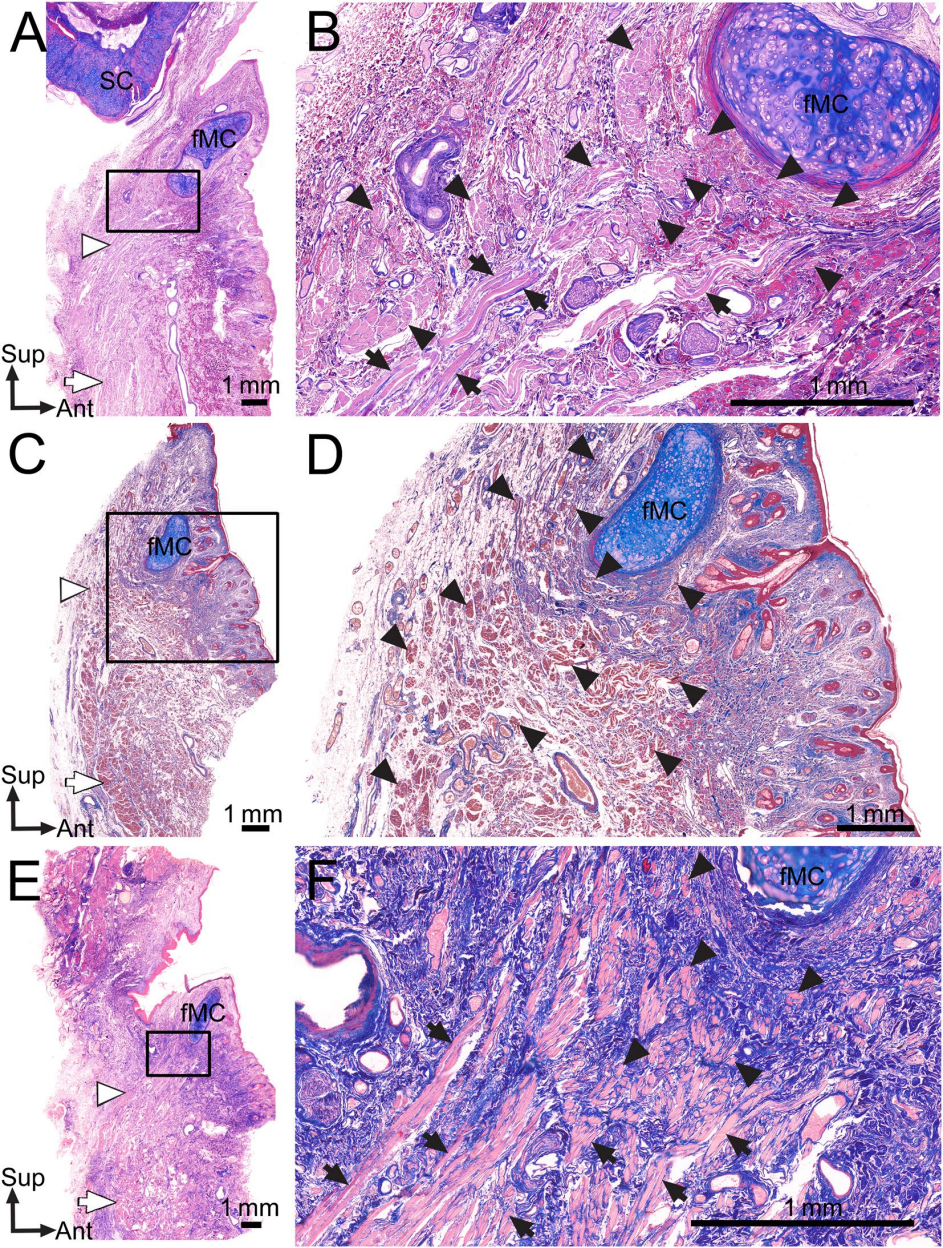
Sagittal sections of histological examinations of the muscle fibers attached to the fMCs. (**A**,**C**,**E**) In sagittal sections at the fMC level, the fMC was found just below the skin. Some muscle fibers were found below the fMC. The originating DSN fibers (white arrows) were thicker than its inserting fibers (white arrowheads). (**B**) Enlarged view of the rectangular area in panel A. Some muscle fibers attached to and adjacent to the fMC were found just posteroinferior to the fMC, coursing medially (arrowheads) and anterosuperiorly (arrows). (**D**) Enlarged view of the rectangular area in panel **C**. Some DSN fibers (arrowheads) were found just posterior to the inferior aspect of the fMC and posteroinferior to the base of the fMC. The muscle fibers of the DSN usually coursed obliquely and medially. (**F**) Enlarged view of the rectangular area in panel **E**. The muscle fibers of the DSN (arrows) usually ascended anterosuperiorly. Some muscle fibers (arrowheads) coursed medially or obliquely adjacent to the fMC. *Ant* anterior, *Sup* superior.

Sagittal sections of the nose obtained at several levels of the fMCs indicated that the muscle fibers located posteroinferior to the fMCs were arranged transversely, obliquely, and anterosuperiorly. The DSN had thicker originating than inserting fibers (Fig. 5). Since the crossing fibers ascended obliquely and medially toward the fMC on the contralateral side, the crossing fibers just posterior to the fMCs appeared as cut fibers in both the transverse and sagittal sections.

## Discussion

The present study is the first to identify several anatomical features of the muscles attached to the fMCs of the major alar cartilage. Our results provide novel insights into anatomical mechanisms that pull the nasal tip downward and shorten the upper lip during smiling or speaking. Specifically, we have found that the DSN and OOr are attached to the mobile fMCs but rarely to the rigid nasal septum. When the DSN is absent, some OOr fibers are attached to the fMC instead of the DSN. Sinno et al.^3^ suggested naming the DSN and OOr as the fMC depressor, which is supported by the results of the present study.

**Figure 6:**
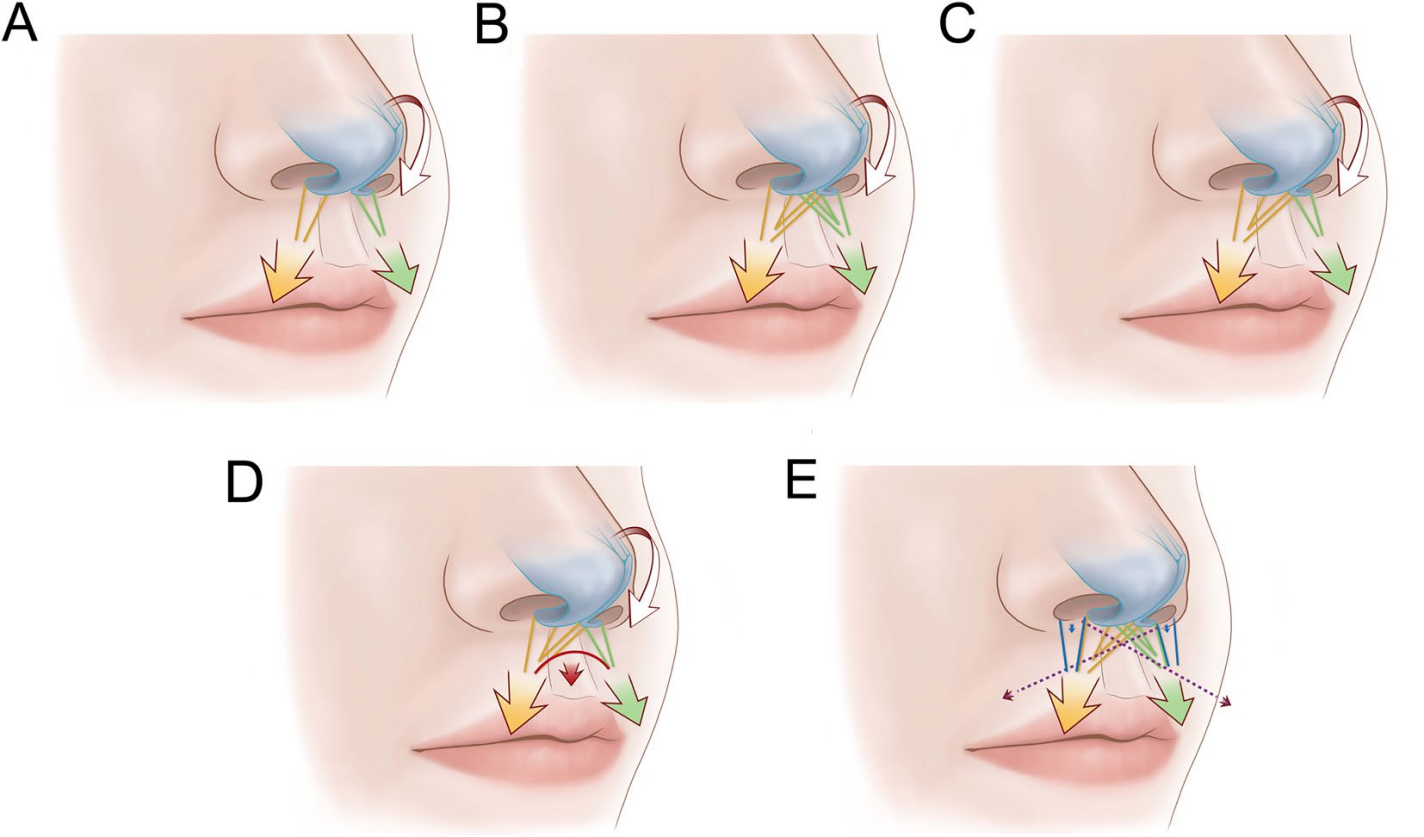
Proposed mechanism of dynamic pulling forces of the muscles attached to the fMCs. (**A**) Contractions of the DSN and OOr (yellow and green) attached to the bases and posterior surfaces of the fMCs may rotate fMCs and cause the nasal tip downward. (**B**) The crossing fibers of the DSN and OOr attached to the fMCs can increase the length of the fibers pulling the fMCs and assist in changing the pulling direction toward the nasal tip. Bilateral crossing fibers of the DSN and OOr could pull the nasal tip downward in a more-medial direction. The crossing fibers of the DSN and OOr attached to the fMCs and their adjacent vestibular skin may increase tension in the nasolabial area below the fMCs, causing the nasal tip to droop and the upper lip to shorten. (**C**) Distinctive unilateral crossing fibers may cause imbalance of the pulling force toward the nasal tip. (**D**) The unilateral crossing fibers of the DSN were often observed with connecting fibers (red) in an arched shape between both the DSNs. These connecting fibers may modify the downward force in a more-medial direction, reducing asymmetric forces on the nasal tip. (**E**) When the DSN and OOr with crossing fibers attached to the fMCs pull the fMCs and nasal tip downward, the depressor alae nasi muscle (blue) located lateral to the DSN may pull the vestibular skin of the nostril sill adjacent to the fMCs downward. The crossing fibers of the OOr attached to the vestibular skin adjacent to the fMCs may assist in pulling the nose downward (purple). Yellow and green arrows indicate directions of the contraction forces of the DSN and OOr. White and red arrows indicate the pulling direction toward the nasal tip. Blue arrows indicate directions of the contraction forces of the DAN. Purple arrows indicate directions of the contraction forces of the crossing fibers of the OOr attached to the vestibular skin adjacent to the fMCs.

Based on our findings, we hypothesize that the anatomical mechanism underlying the dynamic pulling forces in muscles attached to the fMCs involves the following muscle contractions and their vectors (Fig. 6):

A. Contractions of the DSN and OOr attached to the base and posterior surfaces of the fMCs may rotate fMCs and pull the nasal tip downward (Types IV and VI).
B. The crossing fibers of the DSN and OOr attached to the fMCs can increase the length of the muscle fibers that pull the fMCs and assist in changing the pulling direction to the nasal tip. Bilateral crossing fibers of the DSN and OOr (Types I and II) are predicted to pull the nasal tip downward in a more-medial direction. The crossing fibers of the DSN and OOr attached to the fMCs and their adjacent vestibular skin may increase tension in the nasolabial area below the fMCs, causing the nasal tip to droop and the upper lip to shorten.
C. Distinctive unilateral crossing fibers (Types III and V) may cause imbalance of the pulling force toward the nasal tip.
D. Unilateral crossing fibers, which were often observed in the DSN with connecting fibers in an arched shape between both the DSNs (Type III), may modify the downward force in a more-medial direction, reducing asymmetric forces applied to the nasal tip.
E The DSN and OOr with their crossing fibers attached to the fMCs may pull the fMCs and nasal tip downward, while the DAN located lateral to the DSN may pull the vestibular skin of the nostril sill adjacent to the fMCs downward. The crossing fibers of the OOr attached to the vestibular skin adjacent to the fMCs may assist in pulling the nose downward.

**Figure 7:**
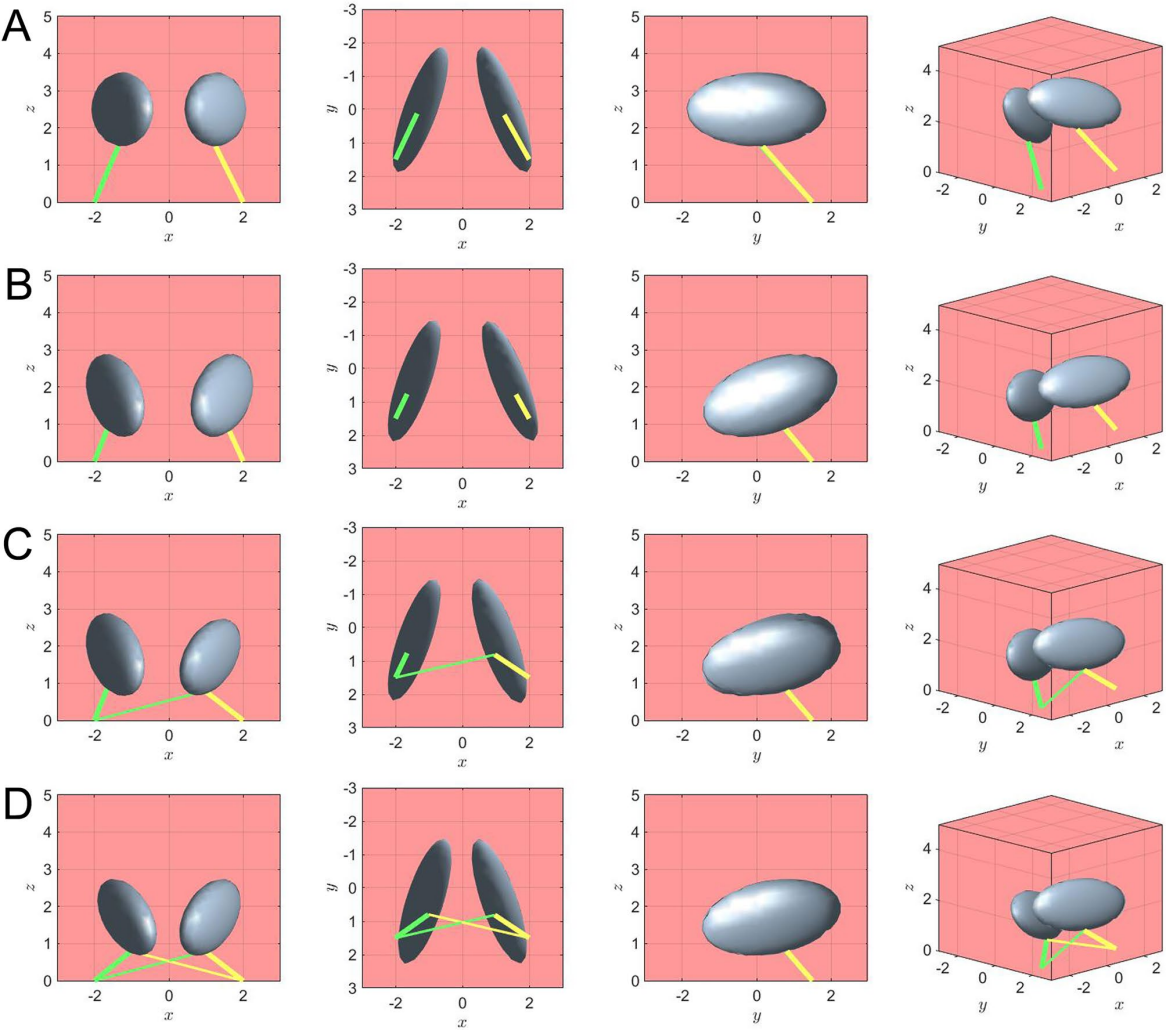
Mathematical simulations of contraction of the DSN or OOr attached to the fMCs (gray ellipses). (**A**) Numerical simulations with an initial condition of the proposed model were performed for three different cases. (**B**) Each DSN or OOr is attached to the fMC on the same side. (**C**) The DSN or OOr on one side has crossing fibers on the contralateral side to the fMC. (**D**) The DSN or OOr has crossing fibers on both sides. For each case, the positions of fMCs and fibers (green and yellow lines) are presented at different angles: front, bottom, side, and oblique views. Note that the main and crossing fibers have different thicknesses. Our results offer support for a possible function of the crossing fibers. The differences between the distances and angles of the fMCs in each case are distinguishable. Moreover, the asymmetric connection pulls only one fMC, which leads to it leaning toward one side. A simulation of contraction of the DSN or OOr attached to the fMCs is presented in Supplementary Videos S5–S7. (Note that all variables are nondimensionalized and the computational domain is [− 2.5, 2.5] × [− 3, 3] × [0, 5]).

In our study we simulated contraction of the DSN or OOr attached to the fMCs using a computational and mathematical model. Net forces from pulling forces generated by the muscles were compared with those currently known of the DSN (Fig. 7 and Supplementary Videos S5–S7). Our results offer support for a possible function of the crossing fibers. The differences of distances and angles between fMCs were distinguishable between the cases. Moreover, the asymmetric connection pulls only one fMC, which leads to the fMC leaning toward one side.

Facial asymmetry can be caused by a change in muscle function on one side of the face relative to the other side, and this can have direct effects on facial expressions^14–16^. Significant facial asymmetry often leads to not only functional but also esthetic issues. Under these conditions, the etiology of facial asymmetry should be carefully investigated to achieve an adequate treatment plan for patients^17^. Contraction of the unilateral crossing fibers in the present study may produce structural and functional asymmetry of the nasal tip, which can also be one of the etiologies of facial asymmetry during facial expression.

The origin and insertion of the DSN are controversial, with some authors reporting the DSN as originating at the maxilla and inserting into the fMC, anterior nasal septum, membranous septum, or columella^12,18–21^, with others state that the DSN originates at the fMC or caudal septum and inserts into the OOr, maxilla, or incisivus labii superioris^5,6,11^. Daniel et al. (2013)^12^ suggested that the origin of the DSN depends on the dissection approach, with the DSN appearing to originate at the maxilla when using an intraoral approach, but at the OOr when using a top-down approach. In the present study, only the OOr was attached to the fMCs instead of the DSN in 20.6% of the samples. If these OOr fibers were defined as the DSN, the origin and insertions of the DSN can be reversely considered such as in the previous studies. Its origin or insertion might therefore be determined according to how the DSN has been defined by different authors. To analyze nasal dynamics in relation to the nasal tip, we focused on muscle attachments to the fMC in this study.

The anatomical variation in muscle morphology that we have observed among individual Korean adult cadavers is striking. Similarly, several anatomical variations of the DSN have been reported among different races and/or ethnic groups. Rohrich et al.^6^ found three anatomical variations in Caucasians: Type I, inserted fully into the OOr (62%); Type II, inserted fully into the periosteum and partially into the OOr from the origin at the fMC (22%); and Type III, absent or rudimentary (16%). Ebrahimi et al.^5^ found three variations in the insertion points of the DSN with origins at the fMC of the alar cartilage and caudal septum in Iranians: Type I, inserted into the periosteum of the maxilla (44%); Type II, inserted into the OOr (39%); and Type III, diminutive or floating (17%). Japanese people have underdeveloped DSNs compared with Caucasians^7^. Barbosa et al.^22^ found that the DSN was absent in 20% of their samples.

Daniel et al.^12^ found that the superficial OOr was divided into the superficial OOr labialis and superficial OOr nasalis. These authors also state that the superficial OOr nasalis, located in front of the fMCs, is a dominant component of the columellar base and primarily responsible for nasal tip depression, whereas the DSN originated at the maxilla with minimal contribution to the columellar base. In the present study, the DSN and some upper OOr fibers were predominantly attached to the base and posterior surface of the fMCs, but rarely to the septum. Therefore we conclude that the superficial OOr nasalis is a primary depressor of the columellar base while the DSN and some OOr fibers are primary depressors of fMCs.

Our study has also found that the crossing and medial DSN or OOr fibers are usually located below the medial base of the fMCs, whereas the lateral DSN or OOr fibers are usually located below the lateral base of the fMCs. Excision or modification of the muscle fibers below the medial base of the fMCs can cut the crossing or medial DSN or OOr fibers, whereas excision or modification of the muscle fibers below the lateral base of the fMCs can cut the lateral DSN or OOr fibers. Lawson and Reino^23^ reported reduction columelloplasty that involves en bloc removal of soft tissue including the DSN using a full-thickness diamond-shaped excision between the fMCs. This surgical technique may involve resection of the crossing and medial DSN or OOr fibers. Directions of surgical approach to the muscles attached to the fMCs can therefore affect the direction of releasing forces toward the nasal tip.

Darwin^24^ highlighted the importance of comparing facial expressions among humans and other mammals to understand the evolution of non-vocal communication systems. The DSN has been reported to be variably present in *M. mulatta*, *H. sapiens*, hylobatids, and *P. troglodytes*^25,26^, whereas the DSN was present in the majority of the samples of humans that we examined. The DSN attaches from the maxilla to the inferior region of the noses of chimpanzees, orangutans, gorillas, gibbons, siamangs, and bonobos^27–31^. In hylobatids the DSN attaches to the inferior aspect of the nasal septum and to the medial aspect of the upper OOr fibers^26^. In *P. troglodytes* and the rhesus macaque (*M. mulatta*), the DSN attaches superiorly to the skin around the nostril and inferiorly to the OOr^25,32^. Burrows et al.^25^ described that these DSN attachments may function similarly to those in humans; that is, drawing the nasal septum inferiorly. In humans, the DSN pulls the nasal tip whereas the levator labii superioris alaeque nasi elevates the alar base when smiling and laughing, demonstrating coordinated movements together with other facial muscles. Burrow^33^ proposed that the human face may be more specially adapted to emotional communication than any other primate face. The various attachment patterns of the DSN and OOr with their crossing fibers to the fMCs that we have described in the present study lends support to the idea that the movements and facial expressions involving the nose and upper lip are more complicated in humans than what is observed in other primates.

The present study has several limitations. First, under normal conditions, the facial movement produced by contraction of the DSN or OOr depends on both the superior and inferior attachments. However, the samples that we examined were removed from their bones, and thus our observations only pertain to the nasal attachments of the DSN or OOr. Moreover, we did not describe the inferior attachments to the maxilla or soft tissues. Second, the computational model that we developed only predicts the contraction of the DSN or OOr and movement of the fMC in a simplified way that is based solely on the anatomical data from the present study. For the model, the fMC was treated as a free body, although in actuality it is part of a larger cartilage and attached to a variety of soft tissues. In addition, for the purposes of the model, we assumed that the DSN or OOr fibers were contracting in isolation. Thus, our model does not take into consideration the motion of the nasal tip and upper lip. Nonetheless, we believe that the model we have generated provides useful information for our overall analysis and can help make predictions about the possible function of the crossing fibers, which in turn adds support to our results and conclusion.

## Conclusion

This study used microdissection, histology, and micro-CT to identify the attaching patterns of the DSN and OOr with their crossing fibers with the fMCs. Our results and the accompanying numerical simulations may facilitate future evaluations of the dynamic pulling forces of the muscles toward the nasal tip. Overall, the data obtained provide novel insights on the anatomical mechanisms underlying nose dynamics and may also likely inform specific guidelines for surgical treatment of the DSN and OOr attached to the fMCs during rhinoplasty. Our results also provide an anatomical basis for the individual and evolutionary variation observed in human facial expressions, which remains a key question in facial expression research^34–37^.

## Materials and methods

### Samples and dissection

The DSN and OOr attached to the fMCs of the major alar cartilage were investigated in 76 samples from 38 embalmed Korean adult cadavers (20 males, 18 females; mean age 70 years, age range 33–93 years). The muscle fibers attached to the nose in 34 of 38 cadavers were dissected. The noses of all samples were removed en bloc with the septal nasal cartilage and the muscles attached to the facial bones. The inner surfaces of the removed noses and their attached muscles were carefully dissected under a surgical microscope (OPMI 1FC, Carl Zeiss, Oberkochen, Germany). The muscle fibers attached to the fMCs were revealed by cutting the inferior part of the septal nasal cartilage. When the DSN and OOr were observed with crossing fibers attached to the fMCs or nasal septum, these fibers were followed to identify their arrangements, courses, and attachments.

All cadavers were legally donated to the Catholic Kwandong University College of Medicine, and this study was conducted in accordance with the Declaration of Helsinki. No transplant donors were from a vulnerable population and all donors or their next of kin volunteered written informed consent. This study was approved by the Institutional Review Board of the Catholic Kwandong University (IRB No. CKU-21-01-0303).

### Staining for histological analysis

Histological analysis using Masson’s trichrome was performed on the noses of 4 of the 38 cadavers. Our Masson’s trichrome protocol followed; 1. Deparaffinize sections and bring to water. 2. Remove mercury pigment by iodine, sodium thiosulfate sequence. 3. Wash in tap water. 4. Stain nuclei by the celestine blue-hematoxylin method. 5. Differentiate with 1% acid alcohol. 6. Wash well in tap water. 7. Stain in acid fuchsin solution a, 5 min. 8. Rinse in distilled water. 9. Treat with phosphomolybdic acid solution b, 5 min. 10. Drain. 11. Stain with methyl blue solution c, 2–5 min. 12. Rinse in distilled water. 13. Treat with 1% acetic acid, 2 min. 14. Dehydrate through alcohols. 15. Clear in xylene, mount in permanent mounting medium^38^. For histological evaluation of the arrangements of the muscle fibers attached to the fMCs, the samples were stained on 5-μm-thick sagittal and transverse sections (two of each type).

### Analysis of the DSN and OOr using micro-CT images

Four noses from 38 cadavers were dehydrated in 30%, 50%, and 70% ethanol solutions, and were then placed in a 1% phosphotungstic acid solution with 70% ethanol for 8 ~ 12 weeks. Samples were scanned using a micro-CT scanner (Sky Scan 1173, Bruker, Kontich, Belgium) that produces images with 22.40 μm^2^ pixels. Scans were performed on one and three noses after dissection and before histological sectioning, respectively. Three-dimensional (3D) volume-rendered images were analyzed using CTVox software (version 2.7, Bruker).

### Numerical simulations of DSN contraction

We modeled the interaction between fMC motion and the contraction and relaxation of connected fibers in 3D space. Rigid-body motion was modeled using a penalty immersed boundary method described by Kim and Peskin^39^ and a moving overset grid method proposed by Lee et al.^40^. However, the model that we employed was much simpler to implement by requiring only structure–structure interactions, with no fluid–structure interactions. The equations of fMC motion interacting with connected fibers in a 3D space were described as follows:

1 $$ {\mathbf{F}}_{i} \left( {s,\,t} \right)\, = \,K_{i} \,\left[ {{\mathbf{Y}}_{i} \left( {1,\,t} \right)\, - \,{\mathbf{Y}}_{i} \left( {0,\,t} \right)\, - \,\frac{1}{2}\left( {{\mathbf{Y}}_{i} \left( {1,\,0} \right)\, - \,{\mathbf{Y}}_{i} \left( {0,\,0} \right)} \right)} \right], $$

2 $$ {\mathbf{U}}_{{{\text{cm}}}} \left( t \right)\, = \,\frac{{{\text{d}}{\mathbf{X}}_{{{\text{cm}}}} \left( t \right)}}{{{\text{d}}t}}\, = \,\frac{1}{M}\sum\limits_{i} {\int {{\mathbf{F}}_{i} \left( {s,\,t} \right){\text{d}}s} } , $$

3 $$ {{\varvec{\Omega}}}\left( t \right)\, = \,\frac{{\int {\left( {{\mathbf{X}}\left( {s,\,t} \right)\, - \,{\mathbf{X}}_{{{\text{cm}}}} \left( t \right)} \right)} \times \sum\nolimits_{i} {{\mathbf{F}}_{i} \left( {s,\,t} \right){\text{d}}s} }}{{\int {\left\| {{\mathbf{X}}\left( {s,\,t} \right)\, - \,{\mathbf{X}}_{{{\text{cm}}}} \left( t \right)} \right\|_{2} } {\text{d}}s}}, $$

4 $$ \frac{{\partial {\mathbf{X}}\left( {s,\,t} \right)}}{\partial t}\, = \,{\mathbf{U}}\left( {s,\,t} \right)\, = \,{\mathbf{U}}_{{{\text{cm}}}} \left( t \right)\, - \,C\left( {{\mathbf{X}}\left( {s,\,t} \right)\, - \,{\mathbf{X}}_{{{\text{cm}}}} \left( t \right)} \right)\, \times \,{{\varvec{\Omega}}}\left( {s,\,t} \right). $$

where ***F***_*i*_*(s*,*t*) is the spring force generated by the *i*-th connected fiber with parameter *s* defined in [0,1], *K*_*i*_ is the spring-force coefficient, ***Y***_*i*_(*s*,*t*) is the connected-fiber displacement, ***X***_cm_(*t*) and ***U***_cm_(*t*) are respectively the center of mass of the fMC and its velocity, *M* is the mass of the fMC (which was assumed to be one in our simulations), ***Ω***(*t*) is its angular velocity, **X**(*s*,*t*) is its displacement, and *C* is the coefficient of angular momentum.

Simple descriptions of Eqs. (1), (2), (3) and (4) are as follows: (1) represents a pulling force between connecting parts by each *i*-th fiber, with the effect depending on the structure connecting the fibers, (2) and (3) calculate the linear and angular velocities (based on rigid-body motion) of the fMC based on the net force derived using (1), and (4) describes the movement of the footplate. For fMC mesh generation, we used the DistMesh algorithm in MATLAB^41^.

### Ethics approval

This study was approved by the Institutional Review Board of the Catholic Kwandong University (IRB No. CKU-21-01-0303).

### Supplementary Information

Supplementary Video S1.

Supplementary Video S2.

Supplementary Video S3.

Supplementary Video S4.

Supplementary Video 5.

Supplementary Video 6.

Supplementary Video 7.

## Data Availability

All data generated or analysed during this study are included in this published article. All relevant data are contained within the manuscript and its Supporting Information files.
