# Supplementary figures and images for: Crossing fibers may underlie the dynamic pulling forces of muscles that attach to cartilage at the tip of the nose
Source: Sci Rep. 2023 Nov 2;13:18948. doi: 10.1038/s41598-023-45781-1 (PMC10622497; doi:10.1038/s41598-023-45781-1)

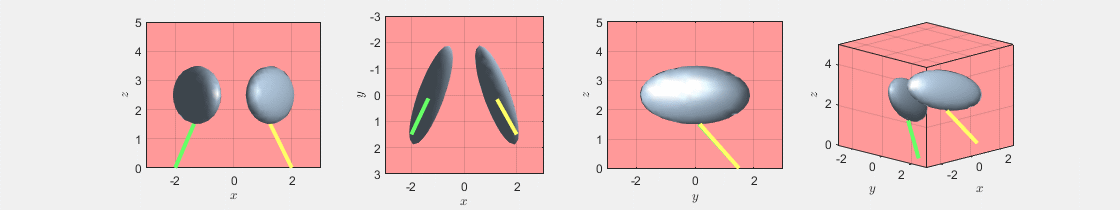

Supplement: Supplementary file 5 — Supplementary Video 5. [file 41598_2023_45781_MOESM5_ESM.gif]

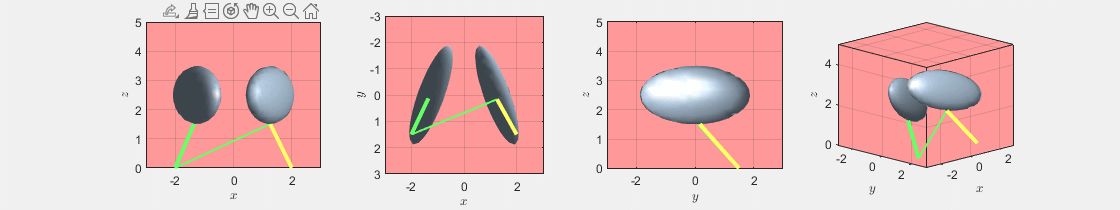

Supplement: Supplementary file 6 — Supplementary Video 6. [file 41598_2023_45781_MOESM6_ESM.gif]

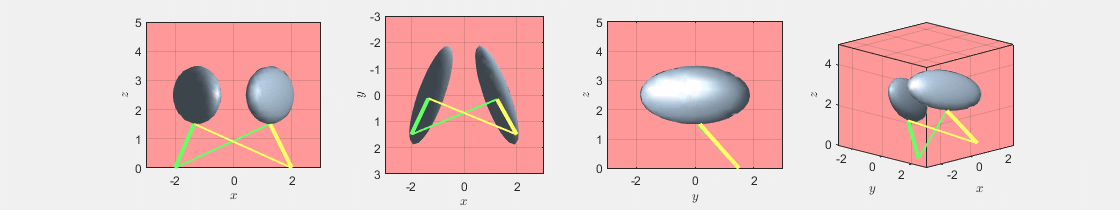

Supplement: Supplementary file 7 — Supplementary Video 7. [file 41598_2023_45781_MOESM7_ESM.gif]
